# Height Error Correction for Shoe-Mounted Inertial Sensors Exploiting Foot Dynamics

**DOI:** 10.3390/s18030888

**Published:** 2018-03-16

**Authors:** Estefania Munoz Diaz, Susanna Kaiser, Dina Bousdar Ahmed

**Affiliations:** German Aerospace Center (DLR), Institute of Communications and Navigation, Oberpfaffenhofen, 82234 Wessling, Germany; susanna.kaiser@dlr.de (S.K.); dina.bousdarahmed@dlr.de (D.B.A.)

**Keywords:** pedestrian, navigation, foot, drift, height, 3D, stairs, horizontal, ramps

## Abstract

Shoe-mounted inertial sensors are widespread deployed in satellite-denied scenarios because of the possibility to re-calibrate the estimated position stepwise. These re-calibrations, known as zero-velocity corrections, prevent an accumulated positioning error growth over time caused by the noise of current medium- and low-cost sensors. However, the error accumulated over time in the height estimation is still an issue under study. The objective of this article is to propose a height correction that is based on the dynamics of the foot. The presented algorithm analyzes the movement of the foot, which is different when walking on horizontal surfaces and stairs. The identification of horizontal surfaces and stairs is detailed in this article. For the assessment of the performance of the proposed height correction, a dataset of approximately 5 h recorded with 10 volunteers walking in a five-story building is employed. The error is evaluated using pre-defined ground truth points. We compare the height error estimated with and without applying the proposed correction and show that the height correction improves the vertical positioning accuracy up to 85%.

## 1. Introduction

There is nowadays an interest in pedestrian positioning systems. Many of them are integrated in safety-of-life services such as disaster management for rescue personnel [1,2,3]. Pedestrian positioning systems, however, are not only restricted to the professional market. Their demand is widespread for all kinds of location-based services such as guidance in airports, hospitals or shopping malls [4].

Most of the aforementioned positioning systems are based on inertial sensors. Typically, inertial sensors, i.e., accelerometers and gyroscopes, are often based on Micro-Electromechanical Sensor (MEMS) technology. MEMS are widely used due to their miniaturization and price reduction. Although many publications have been written about pedestrian inertial positioning systems using medium- and low-cost MEMS sensors, some issues are still under investigation, such as the accumulated error in the height estimation. For example, the authors in [5] apply an empirical threshold to assume that the user is walking horizontally and apply height corrections. These corrections are also applied on stairs. The authors in [6] apply also height constraints based on a finite state machine step detector.

The authors in [7] propose to model the height error using an autoregressive integrated moving average model as part of a simultaneous localization and mapping algorithm. Their results show drift-reduced 3D maps of multi-story buildings based on the repetition of the trajectory, a constant floor separation and a strong structural similarity between different floors.

When not applying a computationally complex simultaneous localization and mapping algorithm including assumptions on the structural similarities of the building, many authors use a barometer that is usually embedded with the inertial sensors [8,9,10]. Barometers use the atmospheric pressure to derive the change in height. Changes in the atmospheric pressure depend not only on the altitude, but also on the season and weather conditions. Therefore, the use of these sensors is challenging, although they are able to help reducing the error in height for shoe-mounted inertial sensors. If the sensor is non-shoe-mounted, in general, the positioning is restricted to 2D because the step-and-heading algorithm is applied instead of the strapdown algorithm. In these cases, the barometer directly provides the height estimation.

The authors in [11] propose the dynamic mapping of the vertical characteristics of a multi-story building based on the extraction of reference pressure during the outdoor-to-indoor transition. They use machine learning to autonomously map the floors of the building with data collected via crowd-sourcing. The proposed algorithm estimates the altitude of each floor and the floor number.

Besides the algorithms based on the barometer sensor, another common approach to solve the height estimation is the use of maps, cameras, global navigation satellite systems or Wi-Fi hotspots, among others [12,13,14,15].

The objective of this article is to present a height error correction algorithm, the Height UPdaTe(HUPT), to efficiently reduce the accumulated error in the height estimation of inertial positioning. The proposed algorithm exploits the dynamics of the foot, being able to seamlessly identify if the user is walking horizontally or climbing stairs. Then, the structure of the building is used to apply height corrections exploiting the fact that the height does not change on horizontal surfaces. The use of the movement of the foot is less prone to errors in identifying different building structures than the already proposed solution of using an empirical threshold based on the erroneous height estimation. The presented algorithm does not require additional sensors, such as a barometer, or additional information, such as maps.

The proposed height error correction is presented in Section 2. First, a brief introduction about inertial shoe-mounted positioning systems is given, and the implementation of the proposed correction in the shoe-mounted positioning algorithm is explained. The identification of different 3D structures based on foot dynamics is also detailed. Section 3 presents the experiments carried out to evaluate the performance of the presented algorithm. Two error figures have been used, namely the error in heading and the error in height. Finally, the conclusions are summarized in Section 4.

## 2. Proposed Height Error Correction (HUPT)

**Figure 1:**
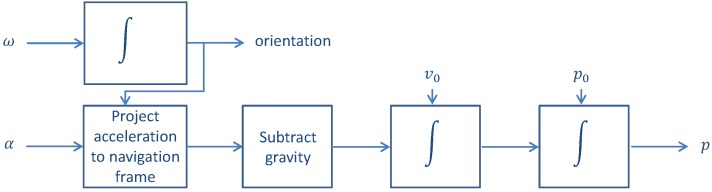
Block diagram of the strapdown inertial positioning algorithm.

The so-called inertial measurement unit contains three mutually orthogonal accelerometers and three mutually orthogonal gyroscopes. Therefore, the acceleration $\alpha =(\alpha_{x},\alpha_{y},\alpha_{z})$ and turn rate $\omega =(\omega_{x},\omega_{y},\omega_{z})$ measurements are triads. For shoe-mounted systems, the strapdown algorithm is usually used to derive the pedestrian positioning. As indicated in Figure 1, the orientation is first computed from the turn rate measurements. The orientation computation is explained in detail in [16].

The direction cosine matrix $C^{k}$ represents the rotation of the body frame b with respect to the navigation frame n of reference at each time *k*: This matrix, $C^{k}$, allows projecting the acceleration measurements $\alpha_{b}$ onto the navigation frame:

(1) $$\alpha_{n}^{k}=C^{k}\cdot \alpha_{b}^{k}.$$

Once the acceleration is expressed in the navigation frame, the gravity $g_{n}$ is subtracted in order to compute the acceleration due to the movement of the body alone. The velocity $v_{n}$ is computed by integrating the acceleration over time, as indicated in the following:

(2) $$v_{n}^{k+1}=v_{n}^{k}+\delta t\cdot \left(\alpha_{n}^{k+1}-g_{n}\right).$$

Finally, the position $p_{n}$ is computed by integrating the obtained velocity over time:

(3) $$p_{n}^{k+1}=p_{n}^{k}+\delta t\cdot v_{n}^{k+1}.$$

The strapdown algorithm is usually implemented through a Kalman filter, where not only the position, but also the velocity and orientation of the sensor, as well as the biases of the inertial sensors are estimated. In order to address the issue of rapidly accumulating errors due to the integrations over time, a re-calibration is applied stepwise. This re-calibration, named Zero velocity UPdaTe (ZUPT), is based on the dynamics of the foot and is therefore only valid for shoe-mounted inertial positioning.

**Figure 2:**
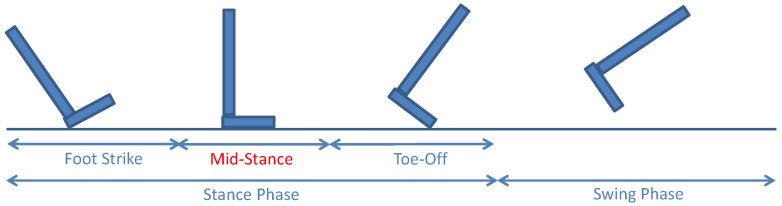
Phases of the gait cycle. During the mid-stance, the Zero velocity UPdaTe (ZUPT) and the Height UPdaTe (HUPT) corrections can be applied.

The gait cycle can be divided into two phases, namely stance and swing. The stance phase applies when the foot is in contact with the floor and the swing phase when the foot is in the air. Within the stance phase, the foot strike, the mid-stance and the toe-off can be identified, as shown in Figure 2.

When the mid-stance phase is detected, the aforementioned re-calibration is applied. The ZUPT correction forces the velocity of the foot, $v^{k}$, to be zero during the mid-stance [17]. Usually, the updates are signals directly measured by the sensors, but also pseudo-measurements can act as updates. We refer the term pseudo measurement in this article to not directly measured signals but assumptions that can be made. The ZUPT correction is a pseudo-measurement $z_{1}^{k}$ that can be written as:

(4) $$z_{1}^{k}={\left[0,0,0\right]}^{T}.$$

There is, however, additional information from the foot that has not been exploited yet. In this article, we propose to identify from the orientation of the foot whether the user is walking on horizontal surfaces or climbing stairs. The proposed HUPT correction forces the *z*-axis position, $p_{z}^{k}$, i.e., the height estimation, not to change when having the certainty that the user is walking on horizontal surfaces and only during the mid-stance. The HUPT correction is a pseudo-measurement $z_{2}^{k}$ that can be written as:

(5) $$z_{2}^{k}=p_{z}^{k-1}.$$

The mid-stance detector required for the novel proposed HUPT is the same used for the ZUPT. The detection is usually performed based on thresholds for the acceleration and turn rate. If both, acceleration and turn rate, are within predefined thresholds during a minimum time, the mid-stance phase is detected. More sophisticated detectors have been proposed in the literature, such as the detection based on a finite-state machine [6].

In order to identify horizontal surfaces and distinguish them from stairs, additional sensors, such as barometers, could be used. In this work, we propose to identify horizontal surfaces and stairs using the orientation of the foot. Particularly, we use the pitch angle estimation.

It is important not to apply the HUPT when walking on ramps. Ramps are, however, not included in this work.

### 2.1. Horizontal Surfaces and Stairs Identification

**Figure 3:**
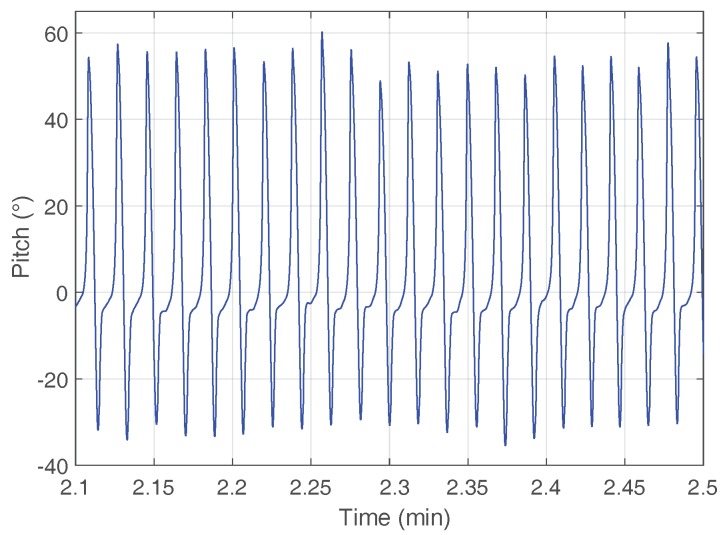
The blue curve represents the pitch angle estimation of a shoe-mounted sensor when walking horizontally. Each maximum of the pitch estimation corresponds to one step.

We propose to use the pitch angle estimation of the shoe-mounted sensor to distinguish between horizontal surfaces and stairs. The foot moves differently when performing different activities. The pitch angle is suited to measure the movement of the foot. Figure 3 shows the pitch angle estimation of a shoe-mounted sensor when walking horizontally.

As the figure shows, if the pitch angle estimation is around zero, the foot is resting on the floor. When the pitch angle estimation increases between 50° and 60°, the foot is under the toe-off phase until the middle of the swing phase (see Figure 2). Then, the pitch angle estimation decreases until −30°, which corresponds to the strike phase. Finally, the foot returns to zero and performs the mid-stance phase.

The movement of the foot described by the pitch angle estimation in Figure 3 is clearly modified during stairs walking. Moreover, the movement of the foot is different when walking upstairs and walking downstairs. A similar behavior has been observed for pocket-mounted sensors, where the movement of the leg is different when performing different activities [18]. Therefore, the pitch angle can be used to enhance the height estimation, as well as for detecting different physical activities.

**Figure 4:**
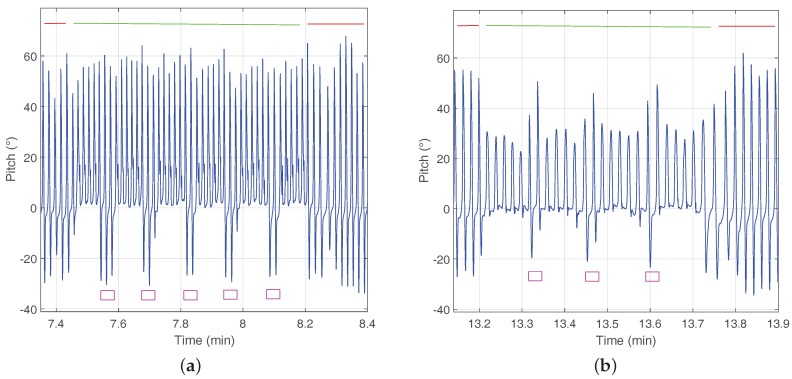
(**a**) Shows in blue the pitch estimation walking down stairs and (**b**) walking up stairs. The red upper line highlights the pitch estimation corresponding to walking horizontally and the green line highlights the pitch estimation walking on stairs. The magenta rectangles highlight the landing zone of the stairs, which corresponds to walking horizontally.

Figure 4 shows the pitch angle estimation corresponding to a shoe-mounted sensor during a multi-story walk. Figure 4a highlights the activity of walking down stairs and Figure 4b walking upstairs. The red upper line corresponds to the user walking horizontally, while the green line corresponds to the stairs.

It is worthwhile to note that, when walking on stairs, the foot strike phase (see Figure 2) does not occur. Instead of this phase, the leg is moving up or down while the foot keeps horizontal, i.e., around zero. The absence of the foot strike phase is the key to distinguishing stairs from horizontal surfaces.

Additionally, walking up stairs can be distinguished from walking down stairs based on the value reached until the middle of the swing phase. When walking downstairs the pitch of the foot reaches higher values during swing phases, that are similar to the maximum pitch values reached when walking horizontally.

The magenta rectangles highlight the short periods of time, usually only a couple of steps, on the landing zone of the staircase. These steps correspond to walking horizontally, as seen in Figure 4.

## 3. Experimental Results

This section is devoted to the experimental results to assess the performance of the proposed height error correction. Firstly, the recorded dataset and the selected error metrics are explained. Secondly, the results are summarized and analyzed.

### 3.1. Data Set and Error Metric

A ground truth point is a point whose location is known accurately. These points are visited during the walk and then the position estimated by the navigation system for these points is compared to their true position. The position of the ground truth points over the five-story building is measured with a tape measurer and a Laser Distance Measurer (LDM). The accuracy of the tape measurer is within the sub-centimeter range, and the LDM measures distances with an accuracy in the millimeter range. In non-line of sight situations, the tape measurer was used to check the measurements given by the LDM.

**Figure 5:**
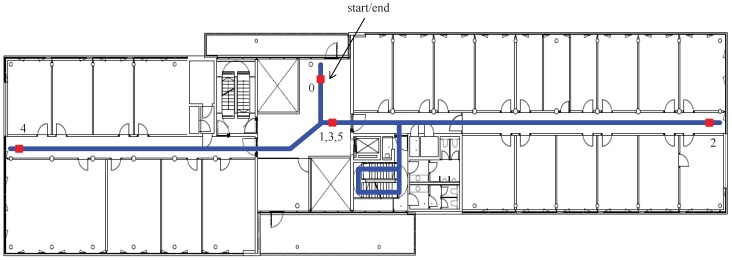
Floor plan of the second floor of the building where the experiments were performed. The trajectory shown in blue is repeated over all floors but the start/end, which is only passed on the second floor. The ground truth points are highlighted in red.

The test building is the Institute of Communications and Navigation of the German Aerospace Center in Oberpfaffenhofen, near Munich (Germany). The length of the building is approximately 68 m, and the width of the building is approximately 17 m. In our experiments, we used three points per floor, at the ends of the corridor and in the middle, and additionally one starting point close to the middle point of the second floor. The final point of the walk is the same as the end point (see Figure 5). A dataset of 3D walks was recorded in order to evaluate the proposed HUPT correction. The trajectory of the walks covers all five floors of the test building.

**Figure 6:**
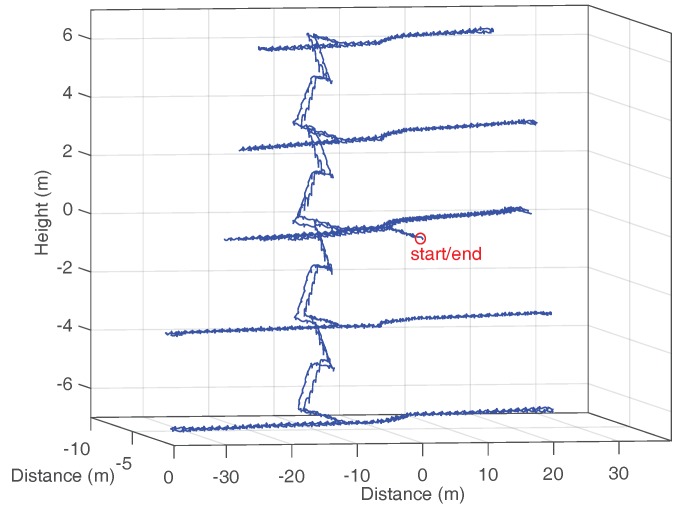
The blue line shows the multi-story estimated trajectory applying the proposed HUPT correction. A red circle highlights the start and ending point.

The walk starts and ends at the same point of the second floor. After starting the walk, the volunteers walk from one end of the corridor to the other in the order indicated by the numbers in Figure 5. Afterwards, the volunteers went to the next floor level where the same routine is repeated, as shown in Figure 6. The floors are visited in the following order: second floor, third floor, fourth floor, ground floor, first floor and second floor. Each walk lasts, approximately, 15 min to 20 min depending on the walking speed of the volunteer. A total of 10 volunteers (3 women and 7 men) participated in the experiments, and each one performed the described trajectory twice. Thus, a total of 20 walks with an approximate overall duration of 5 h comprises the dataset.

During the walk, the volunteers were equipped with an inertial sensor unit placed on the upper front part of the right foot. The sensor used in the experiments is the MTw from Xsens connected to a laptop, and the measurements were logged at 100 Hz. The noise analysis of the three mutually orthogonal gyroscopes of the MTw sensor used for the experiments can be found in [16]. During the walk, the volunteers stopped at the ground truth points for approximately 2 s. These stops are identified afterwards using the norm of the acceleration.

For the performance evaluation, we analyze the 3D positioning error for the proposed HUPT correction. This error can be divided into two, the *x*- and *y*-axes and the *z*-axis error. The error in the *x*- and *y*-axes can be divided into two: error in length and error in heading. Since the inertial navigation system under study is shoe-mounted, we assume no error in the length estimation [19]. Thus, the error in length is not evaluated. The error in the *z*-axis corresponds to the height error. Therefore, we have chosen two error metrics, namely the heading error and the height error.

The error in heading $e_{\psi }$ is computed as the absolute value of the estimated heading of the trajectory $\tilde{\psi }$ when crossing the ground truth point minus the heading of the ground truth point $\psi^{t}$:

(6) $$e_{\psi }=abs\left(\tilde{\psi }-\psi^{t}\right).$$

To compute the error in heading, only the ground truth points situated at both ends of the corridor of all floors have been chosen. The heading associated with the ground truth points $\psi^{t}$ is determined by the direction of the narrow corridor, being 90° and 270° for Points 2 and 4, respectively (see Figure 5).

The error in height $e_{h}$ is computed by the absolute value of the Euclidean distance of the estimated height ${\tilde{p}}_{z}$ at the time the point is crossed to the height of the ground truth point $p_{z}^{t}$:

(7) $$e_{h}=abs\left({\tilde{p}}_{z}-p_{z}^{t}\right).$$

### 3.2. Performance Evaluation

This section summarizes the error evaluation for the proposed HUPT correction. To do that, the positioning error is divided into two: error in *x*- and *y*-axes and error in *z*-axis, i.e., error in height.

#### 3.2.1. Error in *x*- and *y*-Axes

First, we evaluate the error in *x*- and *y*-axes, by using the error in heading $e_{\psi }$. This error is computed according to Equation (6) using ZUPT corrections only and both ZUPT and HUPT corrections. The heading error is evaluated for the 20 recorded trajectories of the presented 5 h dataset.

**Figure 7:**
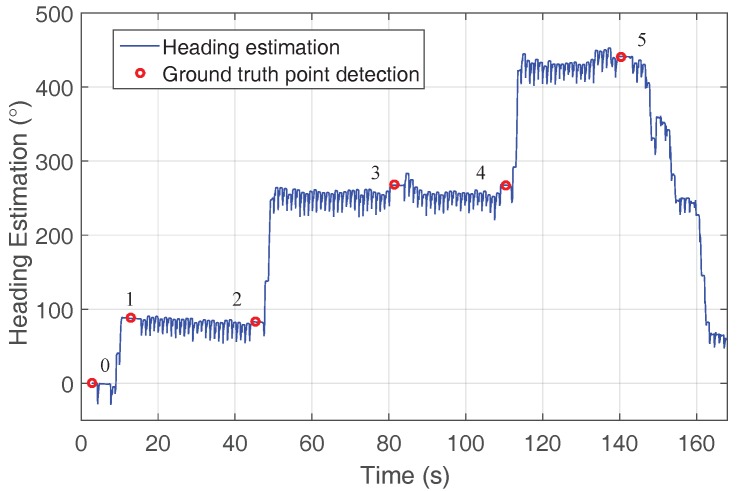
The blue curve represents the heading estimation for one volunteer corresponding to the second floor and the stairs. The ground truth points are highlighted with red circles and labeled, corresponding to Figure 5.

Figure 7 shows the heading estimation corresponding to the beginning of the walk, i.e., second floor and stairs, recorded by one of the volunteers. The automatically-detected ground truth points are highlighted with red circles. These points are labeled corresponding to the points shown in Figure 5.

To compute the heading error, only the points at both ends of the corridor have been selected, e.g., in the case of Figure 5 and Figure 7 for the second floor, Points 2 and 4 are used. Our results show that the heading error differs 0.4° ± 0.7° standard deviation if the error is computed using ZUPT only and using both ZUPT and HUPT. Therefore, we conclude that the proposed HUPT correction has almost no influence on the *x*- and *y*-axis positioning performance of the shoe-mounted positioning system.

#### 3.2.2. Error in *z*-Axis

**Figure 8:**
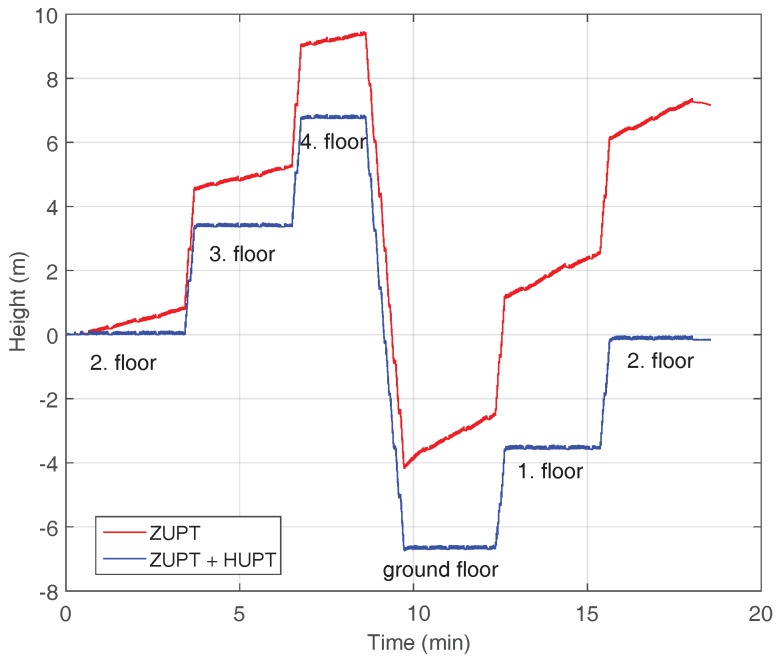
Height estimation corresponding to the walk of one of the volunteers over five floors. The red curve has been computed applying only ZUPT corrections, while the blue curve has been computed applying ZUPT corrections and the proposed HUPT correction.

The error in *z*-axis or height error is computed according to Equation (7) using all ground truth points over the five floors for each of the 20 walks. Figure 8 shows the height estimation corresponding to the walk of one of the volunteers. Since the start and ending point is the same, the final estimated height should end at 0 m. The red curve shows the height estimation using ZUPT corrections only, and the blue curve has been estimated using ZUPT and HUPT corrections.

**Figure 9:**
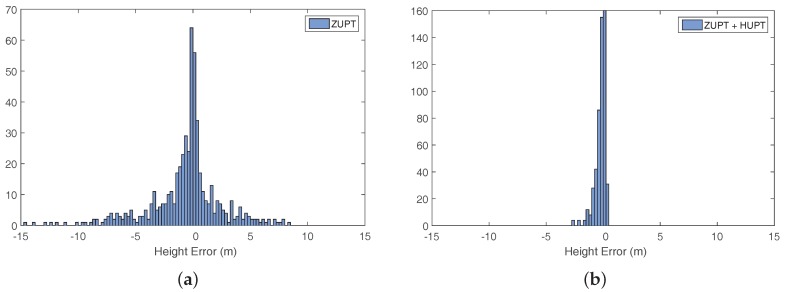
Height error distributions for the evaluated dataset composed of 20 trajectories recorded by 10 volunteers with an overall duration of approximately 5 h. (**a**) shows the result using only ZUPT corrections, and (**b**) shows the result using ZUPT and the proposed HUPT correction.

The evaluation of the error in height corresponding to 534 passes of reference points among all recorded trajectories is presented in Figure 9 using histograms. In order to see the distribution of the error, the sign has been taken into account. Figure 9a shows the distribution of the height error using only ZUPT corrections. Figure 9b shows the distribution of the error using ZUPT and the proposed HUPT correction. The histograms show that the proposed HUPT correction successfully reduces the error in height.

Table 1 summarizes the error computed according to Equation (7), i.e., disregarding the sign of the error. The error is expressed in meters and in the form of the mean error and standard deviation $\left(\mu \pm \sqrt{\sigma }\right)$.

The results show that the mean error can be reduced from approximately 2 m to 30 cm by using the proposed height correction, and its standard deviation is accordingly reduced to ±40 cm. All in all, the proposed height correction is able to improve the *z*-axis positioning accuracy up to 85% in mean and standard deviation.

## 4. Conclusions

The goal of this article is to present a novel algorithm that is able to reduce the well-known error accumulated in the height estimation of shoe-mounted inertial positioning systems. The presented algorithm is based on the dynamics of the foot, i.e., the movement of the foot is different while walking horizontally than while climbing stairs.

The proposed height correction HUPT keeps the previous estimated height for horizontal surfaces only during the phase of the gait cycle in which the height does not change. We prove that the analysis of the dynamics of the foot while walking on different structures significantly improves the height estimation of shoe-mounted inertial navigation systems. The identification of horizontal surfaces and stairs can be done seamlessly without using additional sensors or their erroneous height estimation.

## Figures and Tables

**Table 1:** Error in height $e_{h}$.

	μ (m)	$\sqrt{\sigma }$ (m)
ZUPT	2.10	2.59
ZUPT + HUPT	0.31	0.41
